# Countertransference in the treatment of patients with eating disorders

**DOI:** 10.1186/s40337-025-01439-z

**Published:** 2025-10-27

**Authors:** Almut Zeeck, Vivien Gruteser, Inga Lau, Milena Egenolf, Carolin Klose, Sonja Lienhart, Armin Hartmann

**Affiliations:** Department of Psychosomatic Medicine and Psychotherapy, Center for Mental Health, Faculty of Medicine, University of Freiburg, Freiburg, Germany

**Keywords:** Eating disorder, Countertransference, Emotional reaction, Psychotherapy, Anorexia nervosa, Bulimia nervosa

## Abstract

**Background:**

People with eating disorders (EDs) such as anorexia nervosa (AN) and bulimia nervosa (BN) can trigger intense affective reactions and impulses to act in their counterparts and in those treating them (countertransference, CT). The aim was to summarize the existing evidence on CT in the treatment of EDs by conducting a scoping review oriented on the model of J. Hayes. This model describes CT according to five dimensions: therapist characteristics, triggers, manifestations, effects and management of CT.

**Method:**

The PubMed and EBSCOhost databases were systematically searched for publications on CT in EDs up to October 2024. Studies were screened according to the PRISMA guidelines and findings extracted oriented on Hayes´ dimensions, distinguishing between quantitative and qualitative studies.

**Results:**

From 5346 publications identified, 27 were included in the review (18 quantitative and nine qualitative studies). Even though positive CT reactions predominate, a range of negative reactions (such as helplessness, anger, fear) are described, in particular in the treatment of AN. Therapist variables (professional background, experience), patient´s characteristics (type of the eating disorder, traumatization, personality) and relationship building as well as conditions of the treatment setting are related to the type of CT reactions and its management.

**Conclusion:**

Patients with ED are a difficult group to treat, partly due to the intense CT reactions they can trigger. Therapists should be trained to recognize, mentalize and deal with these reactions and have sufficient time for exchange and supervision during a treatment process.

**Supplementary Information:**

The online version contains supplementary material available at 10.1186/s40337-025-01439-z.

## Abstract (plain language summary)

People with eating disorders such as anorexia nervosa and bulimia nervosa can induce intense emotions and impulses to act in others, including their therapists. These reactions are known as countertransference. The aim of this study was to summarize the results of publications on countertransference in the treatment of eating disorders until October 2024. Out of 5345 publications, 27 were included in the review. Even though positive countertransference reactions seem to predominate, negative reactions (such as helplessness, anger, fear) are frequently described as a challenge when treating eating disorders, in particular anorexia nervosa. The gender and experience of a therapist have an impact on countertransference reactions, as well as the type of patient´s symptoms including her / his personality and symptoms caused by traumatic experiences.

Patients with eating disorders are a challenging group to treat. Intense emotional reactions should be taken seriously, as they may indicate the need for support and supervision.

**Supplementary Information:**

The online version contains supplementary material available at 10.1186/s40337-025-01439-z.

## Introduction

Individuals with an eating disorder (ED) are regarded as challenging to treat [1]. Difficulties in the treatment of EDs include the ambivalence of the patients regarding a change, the self-damaging character of ED symptoms, and complex interactions in relationships, including rejection of the other and secrecy. These may be reasons why individuals’ with an ED were found to induce intense emotional reactions in others – for example anger, fear, and helplessness [1]. The emotional reactions in turn may lead to a strong wish for control, an impulse to take action or overinvolvement [2]. Overall, patients with EDs are treated less readily than other patient groups [3].

Up to now, specialized psychotherapy, regardless of the theoretical orientation, is considered the treatment of choice for EDs [4, 5]. Psychotherapy is an interactional process in which therapists use a treatment model to guide their interventions and behavior, helping a patient recover [6]. However, as psychotherapy research has shown, not techniques as described in a treatment manual are the most relevant components for success, but the quality of the therapeutic alliance and further factors, including those relating to the patient (e.g., motivation, psychological mindedness) and those relating to the therapist (e.g., personality, flexibility, and responsivity) [7]. Against this background, the emotional reactions of therapists in sessions and how these are managed are highly relevant.

In the psychodynamic model, the emotional reactions of a therapist towards a patient are referred to as countertransference reactions. Countertransference (CT) is a concept introduced by Sigmund Freud [8, 9] and further developed and elaborated by psychoanalysts such as Paula Heimann [10]. While an early and more strict definition sees CT as the unconscious response of a therapist to the wishes and feelings the patient projects on him, a broader and more recent conceptualization defines CT as all the reactions of a therapist towards a patient, which are based not only on the wishes, affects and behaviors of the patient, but also the person of the therapist her- or himself, including her / his personality and past relationship experience [9]. The definition of CT has been intensively discussed over time [11, 12]. However, empirical research on this topic is still limited. Nevertheless, reflecting on and being aware of one´s own affective and behavioral reactions as a therapist are relevant for maintaining stable therapeutic alliances [13] as well as for treatment outcome [14].

There is less research on CT compared to other factors relevant to the therapeutic alliance [9], which may be due to different definitions and a lack of operationalization of the concept. In this context, J. Hayes proposed a model to provide orientation for empirical research in this field [15]. His model is currently regarded as the foundation for empirical research on countertransference [7]. Hayes distinguishes (A) “therapist factors”, e.g. personality or unresolved conflicts, (B) “triggers” realized by the patient, e.g. symptoms, interactions or wishes, (C) “manifestations” of CT, e.g. emotional reactions or impulses to act, (D) “effects” of CT on the treatment process, e.g. difficulties experienced by the therapist, and (E) “management” of difficulties, e.g. supervision [15].

Focusing on patients with EDs, there are empirical studies addressing the emotional reactions of therapists or attitudes towards patients. Additionally, there are numerous case reports and theoretical publications, primarily on patients with AN. However, as far as we know, only one review by Thompson-Brenner et al. [1] summarizes this knowledge in English. This review included twenty studies published until 2010. The authors found that negative emotional reactions of therapists in the treatment of EDs primarily include frustration, hopelessness, worry, and a feeling of a lack of competence. Therapist variables such as little experience, gender, stigmatizing beliefs about EDs (e.g. patients with EDs are responsible for their illness themselves), as well as patient characteristics (personality pathology; lack of improvement) were found to predict these reactions. The authors acknowledge that their review included studies conducted at a time when information on EDs was still scarce and efficacious treatments had not yet been established. There is a further review in French [16], which included also theoretical texts and case reports published until 2009. The conclusions of the authors are in line with Thompson-Brenner et al. [1]: Therapists often experience negative affects when treating patients with an ED. These may be induced by the patients themselves, influenced by characteristics of the therapist or related to a lack of knowledge of EDs. Affective reactions may influence the motivation to treat this patient group and quality of treatment. The authors suggest that more empirical research is needed in the field.

 The aim of this study was twofold: First, to update the review by Thompson-Brenner et al. [1] and to systematically search for publications on emotional reactions and countertransference in therapists treating patients with an ED until October 2024. Second, we aimed to structure the findings according to Hayes’ model.

## Methods

### Study design

A systematic literature search was conducted for a recent overview of studies on the emotional reactions (“countertransference” / CT) of therapists working with patients with an ED. The study aimed to gather as much information as possible. Therefore, both quantitative and qualitative studies were included. The content and results of studies were summarized according to Hayes’ model (starting with “manifestations”, e.g., emotional reactions of therapists).

### Literature search

PubMed and EBSCOhost were used as databases (PubMed is one of the largest databases for medical and psychological publications and EBSCOhost an interface for a broad range of literature databases including Medline, CINAHL, PSYNDEX, PsycINFO). The search terms were: “clinician OR therapist OR counselor OR psychologist OR staff OR professional OR provider” AND “reaction OR response OR feeling OR attitude OR emotion OR countertransference OR alliance OR relationship” AND “eating disorder OR anorexia nervosa OR bulimia nervosa OR binge eating disorder”. The search included all publications until October 2024. The aim was a broad initial search. Additionally, publications identified by the reviews of Thompson-Brenner et al. [1] and Forget et al. [25] were included.

In the first step, duplicates were excluded, and titles were screened by one of three authors (VG, AH, AZ), who involved another person in case of uncertainty with the aim to identify all publications that might address countertransference issues, attitudes, and emotional reactions of practitioners in ED treatment. In the next step, abstracts were screened in parallel by two of the three authors (and the decision of in- or exclusion was discussed in cases of discrepancy). Finally, full study texts were retrieved, and further publications were excluded after an independent screening by two authors (AH, AZ). Again, in case of discrepancies, publications were in- or excluded after intense discussion.

### Inclusion and exclusion criteria

The inclusion criteria were: (1) The publication deals with the emotional reactions of professionals working with patients with an ED or their attitude towards them; (2) The publication is available in either German or English language; (3) The patient sample compromises at least 50% of patients diagnosed with an ED (for example when describing emotional reactions of therapists to a group of patients: the majority of patients should have an eating disorder).

Exclusion criteria: (1) Studies on therapist training or training needs of professionals; (2) Publications on theoretical positions or editorials; (3) Publications on specific issues or the therapeutic alliance, which only marginally address aspects of countertransference; (4) Studies on therapists with a “lived experience” of their own ED; (5) Chapters of books or dissertations; (6) Case reports (no empirical study) and theoretical discussions or texts; (7) Studies on very specific and circumscribed topics (e.g., gender minority patients).

It was distinguished between quantitative studies and qualitative studies.

### Extraction of data and content

Two independent raters extracted the data and resolved discrepancies by discussion (AZ, AH). Categories were: therapist characteristics, focus of the study, place of recruitment, patient characteristics (e.g. age, gender, prpfession), measures/instruments used, main results in terms of CT, and additional results. It was coded if the study reported findings related to the dimensions of Hayes’ model [15]. The main essence of the findings was then narratively summarized by two authors (AZ, AH) in a first version, focusing on the model: CT manifestations (affect and impulses to act), triggers on the patient´s side (interpersonal behavior, affect, and symptoms), effects on the therapist´s side, and management of CT.

Finally, the studies were independently reviewed by four additional authors (IL, CK, SL, ME) and the extracted content was re-evaluated. The summaries of the findings were then discussed by the full author group and revised where necessary, for instance, when relevant content had been omitted or findings had not been sufficiently weighted. In the final step, consensus was established.

## Results

**Fig. 1:**
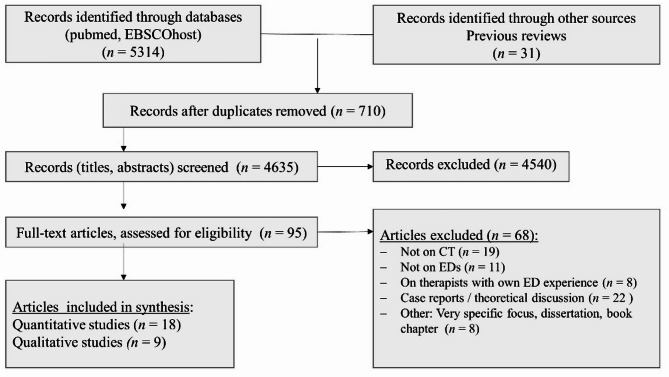
PRISMA-ScR-flowchart

For a PRISMA-ScR-flow-chart see Fig. 1.

Supplement 1 gives an overview and description of included publications on quantitative empirical studies, and Supplement 2 lists studies with a primarily qualitative approach. For studies excluded after the full-text-screening see Supplement 3.

Overall, 18 empirical studies with a quantitative approach and nine qualitative studies were identified. One study combined a quantitative and qualitative approach [17]; it was decided to list it under the quantitative studies. In only five of the 18 studies with a quantitative approach, validated questionnaires were used, such as the Countertransference Questionnaire (CTQ) [18, 19]. The majority of studies, however, were based on self-designed instruments. For a description of the scales of the CTQ, which is also called the Therapist Response Questionnaire (TRQ), and its version for adolescents, see Tables 1 and 2 [19, 20].

**Table 1:** Examples of items from the TRQ – Therapist response questionnaire / CTQ - Countertransference questionnaire (Betan et al. 2005)

Item	Loading
Factor 1 overwhelmed/disorganized (12 items)	
I feel resentful working with him/her.	0.72
S/he frightens me.	0.67
I feel mistreated or abused by him/her.	0.55
Factor 2 helpless/inadequate (8 items)	
I feel I am failing to help him/her or I worry I won’t be able to help him/her.	0.85
I am hopeless working with him /her.	0.78
I feel confused in session with him/her.	0.52
Factor 3 positive (9 items)	
I look forwaqrd to session with him/her.	0.69
I have trouble relating to the feeling s/he expresses.	− 0.47
If she was not my patient, I could imagine being friends with him/her.	0.44
Factor 4 special/overinvolved (5 items)	
I disclose my feelings with him/her more than with other patients.	0.64
I feel guilty when s/he distresses or deteriorates, as if I must be somehow responsible.	0.39
I end sessions overtime with him/her more than with my other patients.	0.39
Factor 5 sexualized (4 items)	
I feel myself being flirtatious with him/her.	0.99
I feel sexually attracted to him/her.	0.89
I feel sexual tension in the room.	0.78
Factor 6 disengaged (6 items)	
I feel bored in session with him/her.	0.82
My mind wanderst o things other than what s/he is talking about.	0.72
I watch the clock with him/her more than with my other patients.	0.46
Factor 7 parental/protective (5 items)	
I feel like I want to protect her.	0.69
I have warm, almost parental feelings towards him/her.	0.67
I feel angry at people in his/her life.	0.45
Factor 8 criticized/mistreated (5 items)	
I feel unappreciated by him/her.	0.75
I feel criticized by him/her.	0.63
I feel I am „walking on eggshells“ around him/her […]	0.56

**Table 2:** Examples of items from the countertransference questionnaire for adolescents (Zittel & Westen 2003; satir et al. 2009)

Item	Loading
Factor 1 angry and frustrated (7 items)	
I feel angry at her	0.78
At times I dislike her	0.67
I dread sessions with her	0.67
Factor 2 warm and conpetent (7 items)	
I have warm, almost parental feelings towards her	0.72
I like her very much	0.70
I feel nuturing towards her	0.68
Factor 3 agressive and sexual (7 items)	
I feel like Iám being mean or cruel to her	0.73
Her sexual feelings towards me make me anxious or uncomfortable	0.65
I feel sexual tension in the room	0.65
Factor 4 faing and incompetent (7 items)	
I feel I am failing to help her or I worry I won´t be able to help her	0.70
I feel lime my hands have been tied or that I have been put in an impossible bind	0.62
I feel incompetent or inadequat working with her	0.55
Factor 5 bored and angry at parents (7 items)	
I feel bored in sessions with her	0.66
I don´t feel fully engaged in sessions with her	0.64
I feel angry at people in her life	0.60
Factor 6 overinvested and worried (6 items)	
I do things for her, or go the extra mile for her in ways that I don´t for other patients	0.66
I end sessions overtime with her more than with my other patients	0.57
I worry about her after sessions more than other patients	0.55

Most studies included psychotherapists, mostly psychologists or psychiatrists; four studies included only nurses, and three studies included other health care professionals such as general practitioners or gynecologists.

### Manifestations

#### Studies with a quantitative approach

In terms of the level of positive or negative affect, studies published after the year 2000 which used a validated instrument such as the TRQ, revealed that the overall level of negative reactions was not high [21–23]; a predominance of positive CT reactions was described (positive, parental/protective, warm/competent). These studies asked therapists to recall particular cases they had treated recently. In line with this finding, Daniel et al. [24], who examined the post-session feelings of therapists treating patients with BN, reported that the majority of therapists experienced positive feelings. In contrast, studies published before 2000 reported more negative reactions and attitudes towards patients with EDs. These studies did not use validated instruments and asked for more general reactions unrelated to case experiences. In a study by Burket & Schramm [25], 54% out of 90 professionals reported empathy and 47% satisfaction when thinking about patients with an ED, but 87% reported frustration, 63% anger, and 47% helplessness. Brotman et al. [26] found a trend of more dysphoric affect and anger towards patients with AN compared to patients with obesity or diabetes; a majority felt distressed and helpless when dealing with AN. Using a self-designed instrument which asks if a person likes to deal with a specific patient group (scale from 1 to 5), Fleming & Szmukler [27] found that patients with schizophrenia (M = 3.16) were significantly (*p* < .001) preferred to patients with an ED (M = 2.83) and these to patients taking recurrent overdoses (M = 2.35). In line with this finding, Sansone et al. [28] reported fewer positive impressions of professionals towards ED patients compared to patients with other (non-psychiatric) diagnoses.

The negative CT reactions described in the studies included being bored, angry, or feeling incompetent [23], feeling frustrated, helpless, or anxious [25], or feeling worried, tired, or sad [17].

#### Studies with a qualitative approach

A majority of nurses and physicians in general practice (*N* = 142/183, 78%; not specialized in the treatment of EDs) reported difficulties in dealing with EDs: they reported feeling helpless and being afraid to offend a patient when screening for the disorder [29]. Ten nurses of a general pediatric ward in Taiwan described feelings of impotence and a lack of confidence when treating patients with EDs, as well as having difficulties understanding the problem [30] and establishing a relationship. Another study analyzing semi-structured interviews with eleven nurses from a general psychiatric ward revealed, as a dominant theme, that nurses reported being overwhelmed by emotion, in particular by conflicting feelings, disappointment, frustration, and incomprehension [31]. Further emotions that were mentioned were sadness, despair, confusion, discomfort, compassion and pity, fear, exhaustion, powerlessness, anger, and anxiety, but also sympathy [31]. This is in line with reports from five nurses working with adolescents with AN on general wards in Australia: they described emotional turmoil, especially frustration and distrust as well as difficulties in establishing a trustful relationship with the patients [32].

In three multidisciplinary teams treating EDs, the work was considered difficult and perceived as frustrating, but only in relation to limited resources [33]. Six “early career” nurses of specialist ED units described the following negative CT reactions in in-depth interviews: anxiety, frustration, feeling in the role of a policeman instead of a nurturing nurse, feeling betrayed as a high investment does not lead to improvement in symptoms, feeling attacked, feeling overwhelmed, but also feeling close to patients who often have the same age, interests, and culture [34]. In a study by Bommen et al. [35], 12 interviews with staff of multiprofessional teams of ED services revealed that participants, who had to deliver treatment against consent felt like punishers instead of helpers and were uncertain if they help or hurt; they felt burdened, sometimes abused or pushed back. Seven therapists treating EDs with AN reported feelings of inadequacy and despair when interventions were ineffective, further on feelings of responsibility, distress, and fear that patients might discontinue treatment [36].

In sum, findings seem to confirm the general assumption that patients with EDs are a difficult group to treat as they may induce – besides positive feelings - a broad range of challenging (negative) emotional responses, primarily related to patients with AN. However, a more negative attitude towards patients with EDs was primarily found by older studies asking more generally about CT feelings and attitudes. In contrast, studies that focus on processes with individual patients using validated instruments provide a more differentiated picture: the majority of CT reactions appear to be positive in the sense of warm and affectionate feelings. However, complex negative reactions may also be present. It must be taken into account that these studies present “snapshots” on individual cases. The kind of negative affect ranges from feelings of helplessness, incompetency or powerlessness to frustration and disappointment; therapists report difficulties in understanding the eating pathology of a patient and feelings of anxiety, boredom or confusion. Furthermore, the patients are reported to induce anger and feelings of being pushed back or betrayed. Positive affects described include sympathy, compassion, pity, and a feeling of closeness and responsibility.

### Therapist’s characteristics

#### Studies with a quantitative approach

*Gender*: Only a few studies explored the impact of gender. Fleming & Szmukler [27] reported no gender differences concerning attitudes toward patients with an ED in a subsample of 163 medical students and physicians (the subsample of nurses consisted almost exclusively of women and therefore did not allow a comparison; whole sample *N* = 352). In two studies studying CT reactions explicitly [21, 23], the study by Satir et al. [23] on 120 therapists treating adolescent patients found that male therapists reported more warmth and competence on one hand, but also more anger and frustration on the other when compared to female therapists. In the study by Colli et al. [21], including 149 therapists treating adult and not adolescent patients, males again reported more hostile feelings, but also more overwhelming and sexualized reactions than females.

Burket & Schramm [25] found that males were more reluctant to treat patients with an ED, and Morgan [37] reported gender differences in etiologic assumptions: females thought more than males that EDs are culturally determined, whereas more males saw EDs as an abnormal behavior in the context of a weak, manipulative or inadequate personality. Additionally, more male therapists than females found BN to be untreatable [37].

*Professional background*: Few studies explored the professional background and the theoretical orientation as possible variables influencing CT. The study by Satir et al. [23] compared psychiatrists and psychologists working with patients with an ED and found that psychiatrists reported a higher degree of anger and frustration as well as aggressive and sexual reactions. A study by Brotman et al. [26] found that, in contrast to residents in medicine, psychiatrists and pediatricians reported feeling sadder towards patients with AN. Furthermore, they found that residents in medicine overall reported less affect compared to psychiatrists and pediatricians, and psychiatrists reported the highest awareness that their reactions might affect clinical care. When comparing trainees in medicine and student nurses with doctors and nurses after examination, Fleming & Smukler [27] showed that trainees liked ED patients more and held them to be less responsible for their illness than more experienced staff.

One study examined the impact of theoretical orientation and psychotherapy training on CT. In a randomized controlled study on treatment for BN, psychoanalytically oriented therapists reported more negative and fewer positive (post-session) feelings toward their patients compared to cognitive-behavioral therapists [24]. In the same study, an interaction between treatment orientation and attachment patterns of the patients with CT feelings was found: Psychoanalytically oriented therapists felt more indifferent/bored with clients with a dismissive attachment pattern compared to cognitive-behavioral therapists, while cognitive-behavioral therapists in comparison to those with a psychoanalytical orientation felt more overwhelmed/moved with preoccupied clients [24].

A study examining the influence of etiologic assumptions found that exposure to biological/genetic explanations for the etiology of EDs leads to a tendency to blame people with AN less compared to exposure to socio-culturally based explanations [38].

*Experience and age*: In the study of Colli et al. [21], more experience was associated with fewer negative reactions, e.g., feeling helpless, overwhelmed, or overinvolved. This findings aligns with the study by Franko & Rolfe [39], which showed that more experience (with EDs and overall) was associated with lower levels of frustration, anger, fear, and tension when treating patients with AN. It was further found that more knowledge of EDs was associated with a higher chance that therapists made a follow-up appointment with a patient [40].

Fleming & Szmukler [27] found no influence of age on attitudes towards patients with an ED, but liking to deal with patients with AN was positively correlated with knowledge.

A further “therapist factor”, the general attitude towards patients with EDs in the sense of wanting or not wanting to treat them, seems to be related to the level of empathy: Burket & Schramm [25] found that if empathy is higher, there more often is a wish to treat them (more empathy: 65% vs. less empathy: 32%).

#### Studies with a qualitative approach

In-depths interviews with six nurses revealed that a young age may lead to feelings of closeness to a patient due to the comparable age, shared interests and cultural similarities [34]. In six semi-structured interviews with medical doctors and psychologists in training (aged 26–34), Toman et al. [41] found that a higher age of the therapist was associated with describing the therapeutic relationship as more distant-confrontative.

In sum, findings suggest gender-specific differences in CT reactions, but the results of the few studies available are heterogeneous and no clear pattern can be described. One study found differences in the etiological assumptions of female and male therapists. It must be taken into account that some of the studies (including the one on etiologic assumptions) were conducted more than 20 years ago and knowledge of the etiology and treatment of EDs has increased over the last few decades. Professional background and training appear to influence CT reactions; professionals with a training in treating mental disorders seem to have a stronger awareness of their CT reactions. One study suggests a possible influence of the therapeutic orientation and CT reactions.

There are only a few findings concerning the influence of the therapist’s age; a young age may lead to a closeness to patients of the same age that can blur the line between friendly and professional contact.

### Triggers (patient variables)

#### Studies with a quantitative approach

*Symptomatology (ED diagnoses and symptoms)*: Results of studies show differences in CT reactions between ED diagnoses. However, the findings again are contradictory. Satir et al. [23] report more therapist feelings of warmth and competence towards adolescent patients with AN compared to patients with EDs not otherwise specified (EDNOS) and more feelings of warmth and competence towards patients with EDNOS compared to those with BN; furthermore, therapists reported more feelings of failing and incompetence when treating patients with EDNOS compared to AN, with which they were less overinvested and had fewer worried feelings. Somewhat in contrast, Colli et al. [21] found that treatments of adult patients with criteria of AN are associated with more criticized/mistreated, helpless/inadequate, overwhelmed, special/overinvolved, and disengaged CT reactions compared to treatments of patients with BN criteria (for a description of factors of the TRQ see Table 1). In treating patients with BN, the work was described to be associated with positive, parental, and overwhelmed reactions. In contrast, in patients fulfilling the criteria for EDNOS, CT reactions were similar to those when treating AN (with additional feelings of sexualization).

Franko & Rolfe [39] found more negative reactions towards patients with AN compared to BN: Therapists reported feeling less connected to patients with a diagnosis of AN compared to BN or depression and a trend to feel less successful with patients with AN than with the other diagnostic groups. Additionally, compared to depressive patients, they felt less engaged with patients with AN, and compared to BN, they felt more frustrated (including fear, anger, and tension) and more hopeless [39].

In a study examining the relationship between therapeutic alliance, motivation and CT reactions in patients with AN, a higher motivation to change, as perceived by the therapists, was associated with fewer hostile and helpless CT reactions and more positive feelings, leading to a stronger therapeutic alliance [42].

*History of trauma*: Two studies described traumatization in patients with an ED as a relevant trigger of CT reactions. One study found that in 104 therapists of various orientations, special/overinvolved CT was related to more severe childhood trauma, such as the separation of parents or experiences of violence [22]. In the study by Colli et al. [21], who examined reactions to different aspects of patients’ psychopathology separately, a history of sexual abuse was associated with more protective but also sexual feelings of therapists. Self-harm, which is often a symptom in traumatized patients, was found to trigger a range of CT reactions: therapists felt more overwhelmed/disorganized, special/overinvolved, and also parental/protective. Dissociative symptoms led to feelings of being criticized/mistreated, overwhelmed/disorganized, special/overinvolved, helpless/inadequate, sexualized, and disengaged [21].

*Personality factors*: Two studies investigated therapist responses to patients’ personality styles. Both found that comorbid personality disorders and personality strongly influence CT reactions. Colli et al. [21] even stated that therapist responses were more related to patient personality than ED symptoms. The authors compared three personality styles in terms of CT reactions: A dysregulated style was associated with feeling more mistreated, criticized, overwhelmed, and overinvolved; an over-controlled personality style was associated with feeling disengaged and helpless and a high-functioning style with positive CT reactions.

Satir et al. [23] reported more negative reactions towards patients with a comorbid cluster B or C personality disorder (PD), especially in the group of patients with an EDNOS diagnosis. A dysregulated/constricted PD style was associated with more feelings of anger and frustration and less feelings of warmth and competence. A depressed/inadequate PD style was associated with less aggressive and sexual CT reactions.

*Course of treatment, treatment variables*: 

Treatment outcome and duration of treatment seem to influence CT reactions. Satir et al. [23] found that feelings of failure and incompetence were associated with shorter treatment duration and less positive outcomes. At the same time, improvement correlated with a higher level of aggressive and sexual feelings. In the study by Colli et al. [21], treatment length was positively associated with sexualized and disengaged responses. Furthermore, Hage et al. [43] found that high relapse rates and worries about somatic complications or survival of the patients predicted emotional exhaustion.

Franko & Rolfe showed that higher caseloads (overall and with patients with EDs) were associated with more negative feelings [39]. In terms of the treatment setting, Winston et al. found no difference in attitudes towards patients with an ED (positive vs. negative) when comparing professionals in a specialized versus. a non-specialized center [44].

#### Studies with a qualitative approach

Interviews with nurses in Australian hospitals describing their experience with adolescent females showed that they experienced the interactional behavior of the patients as particularly difficult: they described the patients as lying and deceiving and that they did not trust the relationship with the nursing staff. Furthermore, the nurses stated that the patients often lost weight quickly after discharge and had to be readmitted, which questions the therapeutic investment [32]. Toman et al. [41] differentiated between patients with different BMIs (BMI = body mass index; kg/m²) in BN or the binge-purge subtype of AN and found that the atmosphere when treating patients with a higher BMI was rated as more distant-confrontative. This category entails the dimensions „aggression towards the patient“, „fear of the patient“, „therapist pushes the responsibility for the therapy onto the patient“, „demanding-confronting attitude“, and „emotional distance to the patient“. In patients with a lower BMI, the atmosphere was more often rated as “close-protective” [41], and this was more pronounced in individual therapists compared to group therapists. The “close-protective” category consisted of the dimensions „emotional closeness to the patient“, „anxiety about the patient“, „caring supportive attitude towards the patient“, and „therapist himself feels responsible for the therapy“. It was also found that the atmosphere tended more towards „distant-confrontative“ with a higher age of the patient [41].

Reid et al. [33] interviewed 18 members of three multidisciplinary teams treating EDs and identified the following topics as challenging: the complexity of problems, the heterogeneity of patients, and the need for individualized approaches; treating patients with an ED was considered complex and sometimes frustrating – but only when resources are experienced as limited and the structure of the service as insufficient [33].

In the study by Linville et al. [29] on screening and intervention practices of “frontline medical providers”, 12 in-depth interviews were conducted, including questions on challenges and barriers when working with patients with EDs and assumptions that may hinder adequate screening. Patient-related factors mentioned were a lack of motivation, patient discomfort with treatment, familial denial, and patient relapses [29]. Some participants assume that recovery is not possible, doctors cannot help, and will not be able to change an ED [29]. In ten interviews with nurses of a general pediatric ward, the defensiveness of patients (characterized by a hostile attitude and an unwillingness to open up), a distorted body image, patients’ rigidity and stiffness, as well as the issue of involuntary treatment were described as challenges [30]. Daven et al. [31] identified the following triggers for negative CT reactions: difficulties in understanding the patient, the “shocking” somatic condition, a lack of improvement or relapses, and the demanding behavior of patients. In the study by Ryu et al. [34] with six nurses in specialized ED units, participants described as difficult that the patients do not want to eat (mealtimes were described as an invisible psychological war), and the high sensitivity of patients around the topics food, weight, and appearance, but also the closeness to patients at the same age. 12 interviews with different professionals of a specialized ED service revealed as triggers for difficulties in the treatment with EDs: no commitment of patients to treatment, comorbidity (e.g., traumatization and autism), being confronted with self-harm, suicidality, high mortality rates, problems in the structure of the service or if they had to deliver treatment against consent [35]. In the study by Tragantzopoulou & Giannouli [36], reasons for difficulties in ED treatment as extracted from seven interviews included that patients are perceived as cautious and hesitant to open up, sometimes lying while trying to rely on someone and that it is challenging to achieve trust. Additionally, the interaction with parents is addressed – it may be difficult because patients deny the illness or are hesitant to involve their parents [36].

In sum, the studies indicate that therapists’ CT reactions in the treatment of EDs are multifaceted and shaped by patient variables—including diagnostic groups, trauma history, personality trait and the degree of somatic danger, as well as by treatment-related factors such as duration, outcome, and structural challenges. While some studies highlight more positive reactions (e.g., warmth and competence) towards patients with AN or EDNOS, others underscore a higher prevalence of negative reactions (e.g., feelings of failure, frustration, or disengagement) depending on patient complexity, comorbidities, and perceived motivation. Trauma history and self-harm behaviors further amplify emotional responses, including feelings of protectiveness, being overwhelmed, and even sexualization. Qualitative studies underscore the role of interactional difficulties in inducing negative CT reactions such as defensive attitudes, lack of trust, the ambivalent motivation, and structural barriers (limited resources or involuntary treatment).

### Effects on therapists

#### Studies with a quantitative approach

*Burnout and exhaustion*: Burnout was indicated by therapists in relation to perceiving the personality of patients with an ED as “difficult” and in relation to somatic complications or relapses, but also by a dissonance between felt affects and own expressed feelings [43]. Warren et al. [17] found that negative CT reactions (sadness, frustration, tiredness, concern) are perceived as a burden by 30.3% of ED specialists.

*General attitude*: A wish not to treat a patient with an ED was in 39% of the cases found to be substantiated by CT reactions (39%) and treatment resistance (30%), followed by problems with comorbidity (17%) [25]. Most attendees of an ED Society Meeting (51%) described patients with EDs as more challenging to treat, compared to other mental disorders [3].

*Eating and body perception*: Sansone et al. [28] compared 12 newly hired nurses on an inpatient unit for EDs with eleven nurses of a non-psychiatric unit in terms of mood and eating pathology over of 13 months. They could not find an increased risk for eating or mood disturbance for those working with EDs. In a survey of 71 professionals working with patients with an ED, 28% reported being affected by this work in the sense that they had an increased awareness of food, their physical condition, and their body [45]. Warren et al. [17] asked 43 ED specialists about their experience with patients’ comments about their own appearance. A majority felt or thought that their appearance was being monitored, examined, or evaluated by the patients; some received direct commentaries on it. 70% described a change in their view of food, and half of the therapists reported a change in eating habits as well as increased awareness of the appearance of others. Overall, results from quantitative and qualitative data showed that working with patients with EDs influenced the therapist’s relation to their eating behavior, weight, and appearance [17].

#### Studies with a qualitative approach

The interview study by King and de Sales Turner [32] revealed that nurses, because of their frustration when treating adolescent patients with AN, tended to distance themselves from the patients, spending less time with them. They also described feeing in danger of losing faith in themselves as nurses. The nurses interviewed by Ryu et al. [34] described how the similar age of the patients and shared interests may lead to feelings of friendship and a desire to rescue a patient. Additionally, it was stated that the sensitivity of patients towards specific topics (e.g., food, weight) leads to being very careful when communicating with them (“walking on an eggshell”) [34]. In interviews with seven psychotherapists of patients with AN, participants reported pressure to provide help as well as a pressure to be careful with words and an elevated sense of responsibility [36].

In sum, studies consistently demonstrate that working with patients with EDs impacts therapists and their professional attitudes on multiple levels. Quantitative findings reveal that negative CT reactions (such as sadness, frustration, and tiredness) are experienced as burdensome and that burnout is more likely when therapists perceive patient personalities as difficult or face complex treatment scenarios like relapses and somatic complications. Frustration and feeling helpless or insufficient may lead to distancing. Additionally, therapists report that working with ED patients can alter their own eating habits and body image, as well as evoke self-critical awareness in the presence of patient comments about their appearance. Qualitative data underscore that these effects are not limited to emotional exhaustion but also encompass relational challenges: Therapists may feel pressured to rescue patients or be overly cautious in communication, driven by heightened responsibility and the complexity of establishing trust with this patient group. Altogether, these studies highlight how the unique characteristics of patients with EDs and the intense therapeutic dynamics can profoundly shape therapists’ emotional and behavioral experiences, underscoring the need for reflective practice and support.

### Management

In the study by Warren et al. [17], therapists overall described an impact on affect, cognitions, and behavior through ED patients, and 30.2% of them found that managing negative affect (worry, fatigue, sadness, and frustration) to be challenging. When therapists were asked for their suggestions for CT management, their suggestions were: to attend supervision, seek consultation, acknowledge the seriousness of the illness and be clear that one cannot always be successful; to limit the caseload with EDs; to work in a multidisciplinary team; to be aware of comorbidity; to read new research/materials and/or to engage in self-care and outside social support. In the study by Franko and Rolfe [39], therapists found supervision or consultation with colleagues to be most helpful (98% of all therapists), while some therapists (24%) referred to their level of experience with treating EDs and self-reflection.

#### Studies with a qualitative approach

In the interviews with nurses conducted by Davén et al. [31], the following aspects were described as helpful in managing difficulties and problems in the treatment of EDs on an emotional, cognitive, and interpersonal level: seeking support and safety in the team, trying to understand the illness and gaining more knowledge, routines and treatment program guides, and time to build relationships with patients and parents. This is supported by the interviews conducted by King and de Sales Turner [32], in which stepping back and reflecting on the difficulties as well as an attempt to understand the patients are described as helpful. In the interviews analyzed by Bommen et al. [35], exchange, supervision, being able to express oneself within the team, the rewarding aspect of relational work with the patients over a longer period, and adjusting expectations were mentioned as important factors for coping.

In sum, although most studies on CT reactions do not specifically address how these reactions are managed, the available findings emphasize that supervision, peer consultation, team-based support, and continuous education are vital for managing the intense emotional responses evoked by patients with EDs. These strategies help therapists to process and reflect upon their CT, thereby reducing negative emotional reactions and fostering therapeutic presence and effectiveness. Additionally, understanding the illness, adjusting expectations, and investing time in building therapeutic relationships appear crucial in mitigating CT challenges and enhancing treatment outcomes.

For a summary of all findings see Table 3.

**Table 3:** Summary of the key findings related to each component of Hayes’ model

Manifestations
- In studies published before the year 2000, mostly negative emotional responses (e.g. being bored, angry, or feeling incompetent, frustrated, angry, helpless, anxious worried, tired, or sad) can be found. - In studies published after the year 2000 positive CT reactions (parental/protective, warm/competent) are predominant, but complex negative reactions were also described. The studies mostly focused on processes with individual patients and mostly used validated instruments. - Health workers not specialized in ED-treatment reported negative feelings (e.g. helplessness, impotence, sadness, despair, confusion, disappointment, incomprehension, frustration, exhaustion, fear, feeling betrayed, difficulties in understanding the problem and establishing a relationship, lack of confidence in treatment), but also feelings of closeness, compassion and pity. *References*: Quantitative Studies: [[17, 21–28]; Qualitative Studies: [29–35]
Therapist’s characteristics
*Gender*: - Findings suggest gender-specific differences in CT reactions, but the results of studies available are heterogeneous. - Gender differences in expression of affects, etiological assumptions, willingness to treat patients with an ED, and ideas about treatment outcome were described. - One study found differences in the etiological assumptions of female and male therapists. *Professional Background*: - Few studies explored the professional background and the theoretical orientation as possible variables influencing CT: Professionals with a training in treating mental disorders seem to have a stronger awareness of their CT reactions. In one study psychiatrists reported a higher degree of anger and frustration as well as aggressive and sexual reactions than psychologists. - One study suggests a possible influence of the therapeutic orientation on CT (cognitive-behavioral vs. psychodynamic). - Clinical experience appears to be a relevant factor, protective of negative and dysfunctional CT reactions. - Exposure to biological/genetic explanations for the etiology of EDs leads to a tendency to blame people with AN less compared to exposure to socio-culturally based explanations. - Age was found to influence CT reactions: Young age of the therapist may lead to a closeness to patients of the same age; higher age was linked to describing the therapeutic relationship as more distant-confrontative. *References*: Quantitative Studies: [21, 23, 25–27, 37–40]; Qualitative Studies: [34, 41]
Triggers = patient variables
- Therapists’ CT reactions in the treatment of EDs were found to be shaped by o patient variables including diagnostic group, trauma history, personality trait and the degree of somatic danger o treatment-related factors such as duration of treatment, outcome, and structural challenges (work place). - Findings regarding diagnostic groups were contradictory: Some studies highlight more positive reactions (e.g., warmth and competence) towards patients with AN or EDNOS, others underscore a higher prevalence of negative reactions (e.g., feelings of failure, frustration, or disengagement) depending on patient age, complexity, comorbidities, and perceived motivation. - Trauma history and self-harming behaviors amplify emotional responses, including feelings of protectiveness, being overwhelmed, and even sexualization. - Qualitative studies underscore the role of interactional difficulties (defensive attitude, lack of trust, ambivalent motivation) in inducing negative CT reactions. *References*: - Quantitative Studies: [21–23, 39, 42–44]; Qualitative Studies: [29, 32–36, 41]
Effects on Therapists
Working with patients with EDs impacts therapists and their professional attitudes on several levels: *Burnout and exhaustion*: - Negative CT reactions are experienced as burdensome and burnout is more likely when therapists perceive patient personalities as difficult or face complex treatment scenarios like relapses and somatic complications. *General attitude*: - Patients with EDs were described as more challenging to treat than other mental health issues and negative CT reaction may lead to distancing und reluctance to treat EDs *Eating behavior*,* weight and appearance*: - ED patients can alter therapists own eating habits and body image, as well as evoke self-critical awareness about their appearance. *Communication and Relation*: - Qualitative data indicate that therapists may feel pressured to rescue patients or be overly cautious in communication, driven by heightened responsibility and the complexity of establishing trust with this patient group. *References*: Quantitative Studies: [3, 17, 25, 28, 43, 45]; Qualitative Studies: [32, 34, 36]
Management
- Supervision, peer consultation, team-based support, and continuous education seem to be vital to process and reflect upon CT - In addition, understanding the illness, adjusting expectations, investing time in building therapeutic relationships, working in multidisciplinary teams, being aware of comorbidity, following routines and/or treatment program guides and engaging in self-care were found to be helpful for coping with ED-related CTs. *References*: - Quantitative Studies: [17, 39]; Qualitative Studies: [31–32, 35]

## Discussion

We aimed to narratively summarize the existing evidence on countertransference (CT) in the treatment of patients with EDs, building on an earlier review by Thomsen-Brenner et al. [1]. The findings of the studies were organized according to the model proposed by J. Hayes, which differentiates between therapist factors, manifestations, triggers, effects and management of CT [15]. We identified 27 studies (18 with a quantitative and nine with a qualitative design) focusing on emotional reactions and CT in the treatment of EDs, as well as studies exploring therapists´ attitudes towards this patient group. Fifteen of these studies had already been included in the previous review of Thompson-Brenner et al. [1] and nine in a further review published by Forget et al. in French language [16]. Studies varied in terms of the professional groups studied (psychiatrists, psychologists, nurses and other professionals - specialized or non-specialized), and only a few utilized validated measures. Additionally, studies were published over 40 years (1984–2024), during which knowledge about EDs, treatment approaches and therapist training changed considerably. However, the summary of findings may nonetheless help to identify the most relevant challenges in treating patients with an ED. A variety of factors were found to be related to the type of CT reaction, so the model proposed by Hayes [15] turned out to be helpful in organizing the results.

In terms of “manifestations”, even though positive CT reactions seem to predominate, especially in studies using validated instruments, a broad range of negative emotions (such as feeling frustrated, helpless and incompetent, but also angry or afraid) were described by professionals when treating patients with an ED, especially patients with AN. This is in line with the previous reviews [1, 16]. The emotional reactions described were quite comparable across studies, which were conducted in different countries and health care systems. The patient factors that “trigger” intense emotional reactions in the treatment of EDs can be located in different areas. First, they differ in relation to the type of ED, e.g. between patients with AN and BN and those with a diagnosis of BED or EDNOS. However, in particular, qualitative studies point to the fact that the characteristics of the interaction with patients and relationship building may be crucial in inducing intense emotional reactions. It was repeatedly described that forming a trusting therapeutic relationship was difficult, in particular with patients with AN, and negatively influenced by their ambivalent motivation, their rejection of help, behaviors that undermine treatment and difficulties opening up [46]. The interactions and relationship building were also described as especially difficult in patients with comorbid PDs or trauma experiences. As a trusting therapeutic relationship is a factors associated with a good outcome [47], this finding of specific challenges in alliance building in subgroups of patients with an ED is important and therapists / professionals should be aware of negative CT reactions. Furthermore, negative CT reactions were shown to be associated with more ruptures in the therapeutic alliance and less resolution of those [13]. It is important to note at this point that “positive” CT reactions are not to be equated with “good” and “negative” CT reactions with bad ones. For example, a protective-parental CT may lead to over-involvement and a difficulty in setting boundaries. Anger, on the other hand, may be an indication that a patient does not submit to everything in a positive sense, but takes her own position.

Another factor influencing the emotional responses of therapists was the somatic danger associated with patients presenting with a low BMI. Such a precarious physical condition may elicit an impulse to protect or control the patient, but it can also provoke fear and a heightened sense responsibility. Moreover, the findings suggest that the nature of therapists` emotional reactions may vary according to the patient´s age (child, adolescent patient, adult patient) and may also fluctuate over the course of treatment. These fluctuations appear to be related not only to the degree of clinical improvement, but also – presumably – to the patient´s internal developmental process, which may, for instance, give rise to more aggressive and sexualized reactions in later stages of psychotherapy.

“Effects” of intense and negative CT reactions described in the studies were exhaustion, a general reluctance to treat patients with an ED, or, in single cases, wishes to get rid of a patient or reject her. These findings underline the need for sufficient supervision and support of therapists and treatment teams. Furthermore, dealing with patients with an ED seems to influence on how therapists experience their bodies and reflect on their eating, at least in terms of increased awareness.

In terms of “therapist factors”, CT was found to depend on the role of the therapist / professional and the function she / he has in the treatment of a patient as well as the conditions and structure of the treatment program. Working with a patient with an ED appears to be especially challenging for nurses, who are usually in much closer contact with patients compared to physicians / psychiatrists or psychologists. Nurses are responsible for maintaining the therapeutic milieu and often directly involved in weighing and meal supervision. A lack of distancing options can lead to greater emotional stress and burden, especially when there is less specific training and supervision [48]. Overall, Negative reactions seem to be stronger in less experienced and trained therapists, which points to the need for specific training and education. There also seem to be gender differences in CT reactions. Here, qualitative studies may provide a more in-depths understanding of the so far heterogeneous findings. Overall, no studies were found that examined the influence of therapists’ unconscious conflicts on their CT response – the aspect that Hayes’ model actually refers to as “therapist factor”.

“Management” of CT, especially of intense and negative CT reactions, can be considered of major importance. Management will include distancing from and reflecting on emotional reactions and impulses to act.

In the future, more studies on CT in patients with BN, BED or EDNOS are needed, as well as on male patients with an ED [49]. There is also a need for further studies on therapist factors, which go beyond descriptive ones such as attachment style, mentalizing capacities, anxiety management, unconscious conflicts or personality and how these relate to emotional reactions to patients´ behaviors, emotions and needs. The summary of the studies in this review allows to describe therapists’ general CT responses to the patients with eating disorders, but there is no answer to the question of how the above-mentioned therapist factors influence this response. Finally, studies should examine the influence of supervision and other options to manage CT. A consensus on the definition of CT will be an important prerequisite (do we understand all emotional responses of a therapist as CT or only those, which deviate from the “typical” response? ) [50]. To this day, there are various definitions, and it often remains unclear which one is being referred to. An overview published by Hayes et al. [51], in which the authors describe the most prominent definitions of CT (the classical, the totalistic, the complementary and the relational definition) and discuss their advantages and disadvantages, could serve as a starting point.

Limitations of this scoping review include that there is no quality assessment and no assessment of risk of bias of the studies. It is exploratory in nature, with a narrative summary of findings and there might be a bias by subjective interpretation of studies. Furthermore, there is a potential for publication bias.

Overall, CT can be considered an important source of information about the therapeutic process, the therapists´ situation and the patient´s inner world [52]. When discussing CT in the treatment of EDs, it should be realized that a high percentage of patients shows attachment insecurity [53] and suffers from profound difficulties regulating feelings and relationships [54, 55], which will increase the probability of challenging CT reactions.

## Conclusions

This scoping review highlights the multifaceted and dynamic nature of CT in treating patients with EDs. Therapists are confronted with a spectrum of affective responses - from warmth and empathy to frustration and helplessness - shaped by patient characteristics, treatment challenges, and therapist-specific factors. These reactions can influence the therapeutic process and the therapeutic alliance and may also impact therapists’ own well-being and professional identity. Supportive measures such as supervision and team-based reflection are highlighted as strengthening awareness and preventing dysfunctional CT reactions; however, more empirical evidence is needed.

### Clinical implications

Since patients with eating disorders can trigger strong and challenging emotions in therapists, they should be made aware that this can be understood as an ‘expected normal occurrence’ for which they should not be criticised and for which they need not feel ashamed. Therapists should be encouraged to use their emotional reactions as a ‘diagnostic tool’ that allows to recognize ruptures in the alliance or critical phases of therapy. They should ‘accept’ their emotional reactions and impulses, but not ‘act them out’ (for example, deviate from the usual procedures, grant special privileges or exercise excessive control without reflection). Therapists, teams and supervisors should promote a supportive culture of constructive processing countertransference reactions, for example by allowing sufficient time for supervision and collegial consultation. Specific additions to treatment manuals how to manage CT reactions - tailored to the respective therapeutic approach - may be helpful.

## Supplementary Information

Below is the link to the electronic supplementary material.

Supplementary Material 1

Supplementary Material 2

Supplementary Material 3

## Data Availability

The material is available from the corresponding author upon reasonable request.
