# Supplementary material for: Countertransference in the treatment of patients with eating disorders
Source: J Eat Disord. 2025 Oct 27;13:240. doi: 10.1186/s40337-025-01439-z (PMC12560542; doi:10.1186/s40337-025-01439-z)
Supplement: Supplementary file 1 — Supplementary Material 1 [file 40337_2025_1439_MOESM1_ESM.docx]

| **Supplement 1: Included studies: Quantitative Analyses** | | | | | | | | | | | | |
| --- | --- | --- | --- | --- | --- | --- | --- | --- | --- | --- | --- | --- |
| **Publication** | **Therapists**  **N**  **characteristics** | **Focus of study** | **Place of  recruitment** | **Patients**  **N**  **characteristics** | **CT- Measures**  **Further measures** | **Main results**  **(CT, emotional reactions)** | **Additional results (associated factors):** | **TH characteristics** | **Trigger P** | **Manifestations TH** | **Effects TH** | **Management TH** |
| 1.  Satir et al.,  2009  ##  ** | N = 120  50.8% female  68.3 % psychologists  31.7% psychiatrists  M = 21,7 ys of experience  45,8% CBT 35,0% PD 8,3% biologic  6% family/systems  5% „other“  (40% „eclectic“ or „integrative“ in some regard)  17.5% specialized in ED treatment | CT and factors impacting CT | Practice network  Register of the American Psychiatric and American Psychological Associations | N = 120  Female patients with an ED (last patient treated last week)  Adolescents (15-18 ys, M = 16.5)  ≥ 6 contacts, ≤ 1 y of treatment  15.0 % AN (50% BP, 50% restrictive)  36.7% BN  47.5% EDNOS  0.8% missing data  22.5% history of hospital treatment for ED  19.2% history of hospital treatment for other reasons | Countertransference Questionnaire for adolescents, CQ-A  (86-items)  Score: 1-7  Adolescent Eating Symptom Form  SWAP-200-A  Psychotherapy Effectiveness Form (PEF) | 6 factors identified:  - angry/ frustrated  - warm/ competent  - aggressive/ sexual  - failing/ incompetent - bored/ angry at parents - overinvested/ worried  Overall level of negative reactions: not high  Highest neg. reactions:  -Bored/ angry (2.38)  -failing/incompetent (2.02)  Mostly:  -warm/competent (3.36) | Male > female therapists:  -warmth/ competence  - anger towards parents  Psychiatrists > psychol.:  -anger / frustration  -aggressive/sexual reactions  Warmth/ competence:  AN > EDNOS > BN  Failing/incompetence:  EDNOS > AN  Comorbid PD influences CT (more negative reactions with cluster B/C, esp. EDNOS)  Dysregulated/ constricted PD styles   - more anger/ frustration  - less warmth/ competence   Depressed/ inadequate PD style:   - less aggressive/ sexual reactions  Treatment outcome and duration influences CT (failure/incompetence associated with shorter duration and less positive outcome; aggressive/sexual feelings associated with improvement) | X | X | X |  |  |
| 2  Colli et al., 2015 | N = 149  71.1% female   36.9% psychol.  63.1% psychiatr.  M = 12.1 ys of experience  (min. 3 ys)  47,6%: CBT  35,6%: PP  16,8%: eclectic  A minimum of one case in current treatment | CT and factors impacting CT | Associations of psychodyn. and cognitive-behavioral PT,  Italy  Centers specialized in the treatment of EDs | N = 149  Female patients with an ED (last patient treated)  Adults (M = 25 ys)  Minimum of 8 sessions (length of treatment M = 14 months)  57% private practice  43% institutions  39.6 % AN (44.1% BP, 55.9% restr.)  31.5% BN  28.9% EDNOS  52.3% comorbid PD  GAF M = 58.9  30.2% history of psychiatric hospitalization | Therapist Response (Countertransference)  Questionnaire (TRQ)  8 Dimensions:  -over-whelmed/ disorganized  - helpless/ inadequate - positive  - special/ overinvolved - sexualized - disengaged  - parental/ protective  - critizised/ mistreated    SWAP-200  Clinical Questionnaire – Eating Disorder Form | Diagnosis and CT:  AN criteria: associated with more criticized/mistreated, helpless/inadequate, overwhelmed, special/overinvolved and disengaged CT reactions  BN criteria: associated with more positive, parental and overwhelmed reactions  EDNOS criteria: associated with CT reactions similar to AN and sexualization | Therapist responses more related to patient personality than ED symptoms.  Three personality styles:  -Dysregulated style associated with feeling more mistreated, criticized, overwhelmed and overinvolved  - over-controlled style associated with feeling disengaged and helpless  - High-functioning style: positive CT reactions  Further patient variables/symptoms  -Sexual abuse: protective & sexual feelings  -self-harm: overwhelmed/disorganized; special/overinvolved, parental/protective  - dissociative symptoms: criticized/mistreated, overwhelmed/disorganized, special/overinvolved, sexualized, disengaged  Therapist variables:  -gender: in males more hostile, overwhelming and sexualized reactions  - more experience = less negative reactions (helpless, overwhelmed, overinvolved  Treatment variables:  -Treatment length: pos. associated with sexualized and disenganged responses | X | X | X |  |  |
| 3.  Groth et al.,  2020 | N = 104  94.2% female  M = 43.6 ys od age  Masters or doctoral degree in mental  health (MA, MS, MSW, MHC, PhD, PsyD, Medicine)  M = 16.3 yrs of clinical experience  46.2%: CBT  12.5%: PD  13.5% interpersonal 7.7%: family-sytems  3.8% humanistic/  existential  16.3% other | Impact of traumatization on CT | USA, Canada  (different listservs)  Online-survey | N = 104  One patient (13-18 yrs of age), last patient seen meeting the criteria  95.2% female  Adolescents (M = 16.9 ys)  Minimum of 8 sessions  67.3 % AN  12.5% BN  7.7% BED  12.5% EDNOS  Traumatic experiences: 69,2% (multiple: 43,3%) | Therapist Response Questionnaire (TRQ)  8 dimensions (see above)   Childhood Trauma Questionnaire (CTQ)  + 6 items from the  Childhood Traumatic Event Scale  CPPS-ED  (type of interventions: CBT, PD oder AED/adjunctive ED interv.)  WAI-SRT  (alliance) | Overall level of negative reactions: not high  Highest ratings:  - postive (3.36)  - parental/protective (2.51) | Focus on impact of traumatization  Special/overinvolved CT sig. related to more severe childhood trauma (separation of parents; violence) → trauma as a relevant trigger for CT  Overall: CBT interventions > PD interventions > AED interventions  More PD-interventions when more severe trauma and more severe ED pathology  Therapist factors:  Higher age and CBT-orientation related to less special/overinvolved CT  Higher alliances were associated with more PD interventions and less special/overinvolved CT | X | X | X |  |  |
| 4.  Burket & Schramm 1995  ##  ** | N = 90 / 159  51.0 % female  Age:  24%: ≤ 30 ys  39%: 31-40 ys 22%: 41-50 ys  14%: >50 ys  Clinical experience:  9%: <2 ys  40%: 2-7 ys  51%: >7 ys  40.0% psychologists  42.0% psychiatrists  7% counselling psychology  6% social work  6% nursing  47.0%: CBT  23.0%: PD  20.0% eclectic  10.0% other | Attitudes towards ED patients | USA  One University Department of Psychiatry; one Department of Psychology; one College of Nursing; one Counselling Center; one private psychiatric hospital | Questions related to AN and BN | Not validated questionnaire on therapists attitudes towards ED patients:  -desire to treat ED patients  - reasons for not wanting to work with them  - CT  - prognosis  - treatment modalities used | Reported CT-reactions:  Frustration 87%  Anger 63%  Empathy 54%  Satisfaction 47%  Helplessness 47%  Anxiety 29%  Differences between therapists who want to treat EDs vs. not wanting to treat EDs:  Empathy is sign. higher (65% vs. 32%) | 31.0% did not want to treat patients with an ED (males > females; no differences related to discipline, experience or age)  Reasons for not wanting to treat EDs:  - CT reactions (39%)  - treatment resistance (30%)  - problems related to comorbidity (17%)  - physical problems (9%)  - excessive time demands (4%) | X | X | X |  |  |
| 5.  Brotman et al.,  1984 ##  ** | N = 29  38.0% female  M = 27 yrs of age  First year residents  in medicine (48%), psychiatry (31%) and pediatrics (21%) | Emotional reactions to ED patients | Massachusetts General Hospital;  USA | Hypothetical patients comparing three groups:  AN  Diabetes  Obesity | Not validated questionnaire  Rating of emotional reactions (anger, sadness, helplessness, anxiety, stress); calculation of a „dysphoria score“ | Trend to more dysphoric affect towards patients with AN compared to patients with obesity or diabetes  Anger towards AN group > obesity & diabetes groups  A majority felt distressed and helpless when dealing with AN | Psychiatrists and pediatricians felt more sad towards AN compared to residents in medicine  Residents in medicine overall reported less affect compared to psychiatrists and pediatricians  Psychiatrists reported the highest awareness that their reactions might affect clinical care | X |  | X | (X) |  |
| 6.  Fleming & Szmukler 1992 ##  ** | N = 352  67% female  Two groups (nurses, physicians) - 31.5 % nurses  - 22.4% stud. nurses - 10.8% med. students  - 21.9% doctors  - 13.6% psychiatric trainees  M = 26.8 yrs of age  46.6% had experience with ED patients | Attitudes towards ED patients | Australia,  general hospital setting | Attitudes towards three groups:  - AN or BN  - schizophrenia  - pat. taking recurrent overdoses | Not validated questionnaire including the question, if a person likes to deal with ED patients (rating from 1 to 5; 3 = neutral);  further questions on causation of the illness and treatment effectiveness | Patients with schizophrenia (M = 3.16) were preferred to patients with an ED (M = 2.83) and these to patients taking recurrent overdoses (M = 2.35) | Age, gender or experience with work on a psychiatric unit did not influence results; knowledge on AN was associated with “liking to deal” with this patient group  Suggested causes of EDs: emotional problems > influence of family > the media > self-induced  No change in medical students attitudes towards EDs after an 8 week term in psychiatry  Med. trainees and student nurses liked ED patients more and held them to be less responsible for their illness than did doctors and nurses | X |  | X |  |  |
| 7.  Daniel et al., 2015 | N = 12  (treating between 2-19 patients)  8 PPT therapists 75% female M = 17 yrs of experience  4 CBT therapists  75% female  M = 8 yrs of experience | Post-session feelings in ED-treatnent | Kopenhagen (university-based  psychotherapy research clinic)  RCT on CBT vs. PPT for BN | N = 69  Patients with BN  98.6% female  47.8% in PPT  52.2% in CBT   25.8 yrs of age | Post-session feeling checklist (FWC); three factors (happy/enthusiastic; overwhelmed/moved; indifferent/bored)  Adult Attachment Interview (AAI)  EDE (Eating Disorder Examination)  SCL-90-R | Focus on post-session feelings and their relation to patients´ attachment patterns (first 15 sessions):  The majority of feelings was positive.  Interaction of therapy type and attachment pattern with CT-feelings:  PPT therapist felt more indifferent/bored with dismissing clients; CBT therapists felt more overwhelmed/moved with preoccupied clients | Secure attachment was not associated with more positive or less negative feelings  PPT therapists: more negative and fewer positive feelings compared to CBT therapists | X | X | X |  |  |
| 8.  Franko & Rolfe  (1996)  ## ** | N = 32  68.8% females    31.3% psychiatrists  37.5% psychologists  31.3% social worker  M = 8.75 yrs of experience (7 yrs with patients with EDs)  80% PD  20% PD & CBT | Emotional responses to ED patients and coping | USA, clinicians experienced with ED-treatment | Hypothetical patients comparing:  one patients with AN, one patient with BN and one depressed patient  (age 14-40, no personality disorder, seen for at least 6 months, not on medication)  Ratings related to last session | Visual analogue scale (emotional responses, 9 subscales)  Question on what helps with the emotional reactions | 5 subscales differentiated the groups: more negative reactions to AN compared to BN  AN < BN, Depr. „feeling connected“  AN, Depr. > BN „feeling frustrated“ (including fear, anger, tension)  AN > BN „feeling hopeless/helpless“  AN < Depr. „feeling engaged“  AN < BN, Depr. (trend) „feeling successful“ | More experience (with EDs and overall) was associated with less frustration, anger, fear and tension when treating AN  Higher caseloads (overall and with patients with EDs) were associated with more negative feelings  Most helpful with coping: supervision or consultation with colleagues (98% of all therapists): 24%: experience and self-reflection also helpful | X | X | X |  | X |
| 9.  Sansone et al.  (1988)  ## ** | N = 23  100% females  - 12 (52.2%) nurses, newly hired for an ED-unit  M = 40.0 yrs of age  - 11 (47.8%) nurses as control group (non-psychiatric unit)  M = 33.5 yrs of age | Burden on nurses of an ED-unit | Sycamore Hospital, USA | Patients with ED on an ED-unit  Vs  patients on non-psychiatric units | Impressions of the Patient Population Survey (26 items on how patients are experienced)  +  Attitudes towards Patients Questionnaire (1 item: rating „unfavorable to very favorable)  BDI, EAT  Job Satisfac. Inventory  Admin. monthly for 13 months | Less positive impressions towards ED patients | Nurses on the ED unit:  - less distorted eating attitudes  - greater job satisfaction  - lower weights  Over 13 months:  -progressively less favorable impressions in both groups  - progressively lower job satisfaction  In sum: no increased risk for nurses working on an ED unit |  | X | X |  |  |
| 10.  Morgan (1999)  ## | N = 115  42% females  obstetricians, gynecologists  > 1 yr training exp. | Attitudes of gynecologists towards ED patients | Four teaching hospitals (UK, Australia) | Not defined | Newly designed questionnaire, 26 items (including a question on attitudes towards ED) | Etiologic assumptions:  31% „abnormal behavior in the context of a weak, manipulative or inadequate personality“  27% form of neurotic disorder  18% culturally determined  18% form of neurophysiological disorder | Only 20% confident to diagnose EDs  Several wrong believes on clinical pictures (AN and BN)  Gender difference in aetiologic assumptions:  females > males: culturally determined  males > females: abnormal behavior in the context of a weak, manipulative or inadequate personality  males > females: see BN as untreatable | X |  |  |  |  |
| 11.  Shisslak et al. (1999)  ## | N = 71  81.7 % females  M = 37.2 yrs of age  23.9% psychologists 2.8% psychiatists  4.2% med. doctors  35.2 % nurses 8.5% counselors 7.0% social worker 9.8% nutritionists 8.5% others  M = 3.1 yrs of experience | If therapists are affected by the work with EDs | USA; participants of an eating disorder conference + participants of an eating disorder seminar at a university medical school | Not defined | Self-designed questionnaire, 40 items on weight/dieting history, eating patterns and its change since beginning to work with EDs | 28% reported to have been moderatly to greatly affected by the work with EDs (no difference between normal eaters and professionals with problematic eating behavior)  Not related: length of experience  Effects: increased awareness of food, physical condition, appearance and feelings about one´s body |  |  |  | X |  |  |
| 12.  Winston et al.,  (2007)  ## | N = 512  ? % female  consultants, dietitians, general practitioners, senior post-graduate trainees in medicine and psychiatry | Comparing knowledge and attitudes towards EDs in two centers: with and without specialist service | UK  Leicester (specialist ED service)  &  Nottingham  (no specialist ED service) | Patients with AN and BN | Self-designed questionnaire, 30 items on attitudes towards patients with an ED and knowledge | No difference in attitude (positive vs. negative) between centers  No significant relationship between attitude and knowledge for any of the professional groups | Psychiatrists in training had more knowledge about EDs when working at a center with a specialist ED service | X |  | X |  |  |
| 13.  Crisafulli et al., (2008)  ## | N = 98  (13 men and 5 females with a history of AN were excluded)  100% females  undergraduate nursing students  >18 yrs of age  Randomly exposed to  - a biological/genetic explanation of AN  - a sociocultural explanation of AN | Attitudes towards EDs depending on assumptions on etiology | North Carolina, USA | AN | Characteristics Scale (20 items on personality and behavioral attributes of AN)  Affective Reaction Scale (hypothetical reaction to a person with AN)  Opinions Scale (6 statements about AN)  Behavioral attention scale | Overall, slightly negative perception of AN (no group difference), but in the affective reactions slightly positive (no group difference) | Exposure to biological/genetic explanations lead to the tendency to blame people with AN less compared to an exposure to a sociocultural explanation | X |  | X |  |  |
| 14.  Currin et al., (2009)  ## | N = 82 (75 responded to attitude questionnaire)  53.7% females  100% general practitioners  75,4% worked fulltime | Attitudes and knowlegde and their influence on iterventions in GPs | UK | AN and BN | Self-designed attitude questionnaire  (see Fleming & Smukler 1992)    Knowledge questionnaire  Clinical vignettes (questions on diagnosis and preferred treatment) | Attitudes scale not analyzed in terms of affective responses | Substantial gaps in knowledge; more knowledge was associated with a higher chance that a follow-up appointment will be arranged  Knowledge: no impact of gender or working full vs. part-time  Interventions:  95.2% „help the patients to recognize the seriousness of the illness“  89.2% make a follow-up appointment  41.0% ask the patient to use a food diary  37.3% refer the patient to a specialist ED service | X |  |  |  | X |
| 15.  Warren et al.,  (2009)  ##  ** | N = 43  90.7% female  ED specialists  M = 50 yrs of age  broad range of educational backgrounds (social work, medicine, psychology, nursing, dietary management  Multiple orientations, most adhering to more than one approach (34.9% PD, 39.5% CBT)  6 months to 31 yrs of experience  >30% had own experiences with an ED | Experiences and affective reactions when treating patients with EDs, recommenrations for this work | USA  Participants of Multiservice Eating Disorder Association annual conference | EDs in general | Self-designed questionnaire (patient´s comments on therapist appearance; personal reactions; recommendations for others)  Quantitative and qualitative analyses | There is impact on affect, cognitions and behavior  30.2% described that managing negative affect (worry, fatique, sadness, frustration) is challenging | A majority received direct commentaries on their appearance or felt monitored by patients ; a minority felt uncomfortable discussing this  Working with patients with EDs influences the therapists relation to food, weight and appearance  Suggestions of therapists for management:  - Supervision, consultation  - acknowledge the seriousness of the illness, one cannot always be successful  - limit the caselot with EDs  - work in a multidisciplinary team  - be aware of comorbidity  - read new research, materials  - engage in self-care and outside social support |  | X | X | X | X |
| 16.  Reas et al. 2021 | N = 144  Gender?  36.8% psychologists  13.4% medical doctors  14.8% nurses  11.3% dieticians  11.3% social workers  4.9% physiologists  5.6% others  Experience:  22.5% 1-3 yrs  14% 4-6 yrs  10.5% 7-10 yrs  18.9% 11-15 yrs  15.4% 16-20 yrs  18.2% > 20 yrs | Beliefs and attitudes | Attendees of the Nordic Eating Disorder Society Meeting 2018 (Reykjavik) | Unclear,  AN, BN, BED | Modified ED-version of the revised Illness Perception Questionnaire (IPQ), 10 items; plus questions on “comfort working with” and difficulty to treat EDs | 51% perceived EDs to be more difficult to treat compared to other mental disorders, 38.2% : similarly difficult,  3.5% easier to treat,  7% were unsure | Working with BED was rated to lead to less personal enjoyment compared to AN and BN  56.3% felt most confident to work with AN, 35.4% with BN and 7.6% with BED |  |  | X |  |  |
| 17  Hage et al. 2021 | N = 186  M = 44.5 yrs of age  91.4% female  Setting:  21.0% outpatient  79.0% inpatient  26.3% psychologists / medical doctors  50.0% nurses  23.7% others | Burnout among therapists | 11 specialized ED units in Norway |  | Online-survey;  Maslach Burnout Inventory (MBI) (25 items, 3 scales: emotional exhaustion, depersonalization/cynicism, reduced personal accomplishment);  EDBURN  Job Satisfaction Scale;  QPS Nordic;  ERI;  Frankfurt Emotional Work Scale | Emotional exhaustion:  15.6%  26% explained by ED-specific factors (22% in cynicism, 12% in lack of personal accomplishment) | Overall: low levels of burnout  Not relevant: gender, setting, profession  Predictors of emotional exhaustion:  -patient personality perceived as difficult  -high relapse  -therapist worry about patient´s somatic complications / survival  Emotional dissonance (discrepancy between true feelings and expressed feelings; suppression of emotions) predicted burn out |  | X | X | X |  |
| 18  Lev Ari et al. 2024 | N = 156  M = 43.1 yrs of age  79.5% female  71.8& psychologists  10.3% social worker  8.3% psychiatrists  6.4% other psychotherapists  3.2% rehabilitation psychologists or pediatricians  14.3 yrs of experience (8.9 yrs with treating EDs)  Orientation:  59.6% psychodynamic  16% combination of methods  7.1% dialectical behavioral  6.4% no specific orientation  5.8% structural family therapy  5.1% cognitive behavioral | CT as a mediator of the relation-ship between the motivation to change and the therapeutic alliance | Israel  recovery centers, conferences and associations | Last patient with AN with whom therapists had a session with that week (treatment with a minimum of 10 sessions)  M = 20.6 yrs of age  Duration of illness:  M = 5 yrs  63.5% on medication  49.4% with comorbidity  21.2% started treatment with a BMI < 15 kg/m²  Average number of sessions: 61.5 | Countertransference Questionnaire (CTQ)  Working Alliance Questionnaire (WAI-SR)  The staging Algorithm Questionnaire |  | Six CT reactions were associated with the therapeutic alliance: feeling hostile, helpless, overwhelmed and disengaged (negatively) and positive, parental (positively)  Three CT reactions mediated the stage of change/therapeutic alliance relationship: feeling hostile, helpless and feeling positive:  “The more therapists perceived the patient as willing to change, the less hostile and helpless they felt towards their patient, accompanied by a greater degree of positivity. This resulted in a stronger therapeutic alliance as well” (p 53) |  | X |  |  |  |

M = mean; CT = countertransference; AN = anorexia nervosa; BN = bulimia nervosa; EDNOS = eating disorder no otherwise specified; AN-BP = binge-purging type of AN; CQ-A = Counter-Transference Questionnaire for Adolescents; SWAP-200-(A) = Shedler-Westen assessment procedure (for adolescents); CPPS-ED = Comparative Psychotherapy Process Scale; WAI-SFT = Working Alliance Inventory – Short Form Revised-Therapist version; CBT = cognitive-behavioral therapy; PD = psychodynamic; PPT = psychoanalytic psychotherapy; RCT = randomized controlled trial; SCL-90-R = Symptom Check-List 90-revised; BDI = Beck Depression Inventory; EAT = Eating Attitudes Test; IPQ = Illness Perception Questionnaire; EDBURN = eating disorder-specific contributers to burn-out questionnaire; QPS Nordic = General Nordic Questionnaire for Psychological and Social Factors at Work; ERI = Effort-Reward Questionnaire

## = included in the previous review of Thompsen-Brenner et al. 2012

** = included in the review of Forget et al. 2011
