# Supplementary material for: Countertransference in the treatment of patients with eating disorders
Source: J Eat Disord. 2025 Oct 27;13:240. doi: 10.1186/s40337-025-01439-z (PMC12560542; doi:10.1186/s40337-025-01439-z)
Supplement: Supplementary file 2 — Supplementary Material 2 [file 40337_2025_1439_MOESM2_ESM.docx]

| **Supplement 2: Included studies: Qualitative analyses** | | | | | | | | | | | | |
| --- | --- | --- | --- | --- | --- | --- | --- | --- | --- | --- | --- | --- |
| **Publication** | **Therapists**  **N**  **characteristics** | **Focus of study** | **Place of recruitment** | **Patients**  **N**  **characteristics** | **CT- Measures**  **Further measures** | **Main results**  **(CT, emotional reactions)** | **Additional results (associated factors):** | **TH characteristics** | **Trigger P** | **Manifestations TH** | **Effects TH** | **Management TH** |
| 1.  King & de Sales Turner (2000)  ** | 5 nurses | Experiences when working with adolescent females with AN | General hospitals, Victoria, Australia | AN,  adolescents  female | Open interview (“Please describe what it is like to care for adolescent females diagnosed with AN!”)  Transcripts analyzed according to Colaizzi (1978) | - emotional turmoil  - feeling cheated and deceived  - distrust  - feeling inadequate and like a failure  - sadness  - anger  - being disheartened  - feeling overwhelmed by frustration | Challenge to the core value of being e.g. non-judgemental and caring; losing faith in themselves as nurses  Triggers:  -manipulative behavior, lies  -difficulties in establishing a trustful relationship from the side of the patient  - relapses, lack of success  Effects: distancing from the patients, spending less time with them  Management: stand back and reflect, learning experience over time, efforts to understand the patients |  | X | X | X | X |
| 2.  Toman (2002)  ##  ** | N = 6  100% female  Between 26-34 yrs of age  three individual therapists  three group therapists  (PD orientation)  medical doctors or psychologists in training | Influence of BMI on attitudes and feelings of therapists | Inpatient unit, Switzerland | N = 18  BN and AN-BP  100% female  3 BMI-groups (6 patients in each group):  1) <18.5 kg/m²  (M = 23.8 ys)  2) 18.5 - 25 kg/m²  (M = 6.0 ys)  3) > 25 kg/m²  (M = 33.4 ys)  illness duration 5-15 yrs | semi-structured interviews  with two therapists per patient (individual + group therapist)  rating of feelings and attitudes according to nine predefined categories | Higher BMI → atmosphere more often rated as distant-confrontative  Lower BMI →  atmosphere more often rated as close-protective  More pronounced in individual compared to group therapists | The component distant-confrontative entails the categories „aggression towards the patient“, „fear of the patient“, „therapist pushes the responsibility for the therapy onto the patient“, „demanding-confronting attitude“, „emotional distance to the patient“  The component close-protective entails the categories „emotional closeness to the patient“, anxiety about the patient“, „caring supportive attitude towards the patient“, „therapist himself feels responsible for the therapy“  Influence of age: therapeutic relationship tended towards „distant-confrontative“ in older patients | X | X | X |  |  |
| 3.  Linville et al. (2010)  ## | N = 183  70% female  50% between the age of 26-40 yrs  Yrs of experience: > 5  General and family practice physicians, pediatricians, gynecologists, obstetricians; nurses in general and pediatric practice (not specialized in treatment of EDs) | Screening and intervention practices; training needs | Associations of physicians and nurses in Oregon / USA  (not specialized in treatment of EDs) | Patients with EDs in general | Online-survey with 20 items (self-designed) + interview (subgroup, N = 12)  Qualitative analysis | Feeling helpless  Fear of offending a patient | Majority of participants reported difficulties in the treatment with EDs and avoided screening and asking for an ED  Challenges described: lack of motivation, patient discomfort with treatment, familial denial, lack of provider knowledge, patient´s relapses  Myths / assumptions: mental health practitioners are responsible, EDs are “just a phase”, recovery is not possible, doctors cannot help, patients with EDs also have a personality disorder |  | X | X | X | X |
| 4.  Reid et al.,  (2010)  ## | N =18  % female?  mean age?  Three multidisciplinary teams treating EDs (NHS outpatient eating disorder service; inpatient unit in a NHS hospital; privately funded residential service) | View of professionals on ED services | UK,  Edinburgh | AN  BN | Semi-structured interviews; systematic qualitative analysis  Topics:  - Structural problems in services and training  - Specific needs of patients with an ED | Patients with an ED are considered difficult and the work can be frustrating – but only in relation to limited resources and deficits in the structure of services | Challenging topics:  - Complexity of problems  - Heterogeneity of patients,  - need of individualized approaches |  | X | X |  |  |
| 5.  Wu & Chen 2021 | N = 10  100% female  M = 30.0 yrs of age  Nurses  >1 yr of work experience in the hospital  Have treated at least 1 patient with AN | Perceptions and experiences of conflict situations | General pediatric ward (childrens hospital, Taiwan) | Adolescents with AN with life-threatening conditions | Semi-structured interviews  (content analysis)  Questions on training, care experience, suggestions for the team | Three themes:  1.Difficulties establishing a relationship (being seen as an enemy)  2. Feeling to need more time, difficulty understanding the problem  3. Differences in perceptions (eating, body etc.) - unability to change the mind of the patient  Feelings of impotence, lack of confidence | Challenges / trigger:  -defensiveness of patients (hostile attitude, unwillingness to open up)  - unvoluntary treatment  - distorted body image  - rigidity, stiffness |  | X | X |  |  |
| 6.  Daven et al. 2022 | N = 11  72.7% female  M = 42 yrs of age  M = 12.5 yrs of experience | Nurses´ experiences with AN (focus on feelings) | General psychiatric ward, mid-Sweden | Adult patients with AN | Semi-structured interview  Analyzed according to phenomenological hermeneutics | Three main themes:  -overwhelmed by emotions (incomprehension; conflicting feelings, disappointed/frustrated)  -seeking strength to cope  - trying to build relation (patient, relatives) | Further feelings mentioned: sadness, despair, confusion, discomfort, compassion and pity, deep sympathy, wish to understand, frightening, exhausting, powerlessness, anger, anxiety  Trigger:  -difficulty to understand the condition  - “shocking” somatic situation  - no improvement, relapses  - demanding patients  Management:  -seeking support & safety (team)  -trying to understand the illness, gaining more knowledge  -time to build relationships with patients and relatives |  | X | X |  | X |
| 7.  Ryu et al. 2022 | N = 6  50% female  M = 26.1 yrs of age  Nurses, 4 graduate (first year), 2 postgraduate (second year) | Emotional experiences of early career mental health nurses with EDs | Specialist ED-units, Victoria, Australia | Not specified | One-hour in-depths interview  Analyzed by inductive thematic analysis | Initial tension and intense feelings (when starting work on the ward)  Reported CT-reactions:  -anxiety  -frustration  -helplessness  - feeling in the role of a “policeman” or enforcer instead of a nurturing nurse (“corrupting own values”  -feeling betrayed (high investment/involvement, which does not lead to improvement)  - feeling attacked  -feeling overwhelmed  -feeling close (same age, interests, culture)  - feeling helpless | Trigger:  -patients not wanting to eat  -mealtimes as invisible psychological war  -high sensitivity of patients around weight, food, appearance  -same age group, similarities in interests etc.  Effects:  -feelings of “friendship”  -wish to “rescue” the patient, getting overinvolved  - “walking on eggshells”/ being cautious in the communication |  | X | X | X |  |
| 8. Bommen et al. 2023 | N = 12  ~75% female  25-44 yrs of age  Experience with ED:  22% < 1 yr  22% 1-2 yrs  56% 3-5 yrs  25% psychologists  25% health care assistants  16.7% nurses  8.3% psychiatrist  8.3% dietitian  8.3% occup. therapist  8.3% family therapist | Impact of work with severe and complex EDs on professionals in specialized services (including coping) | Inpatient settings, UK | EDs, not specified | Semi-structured interviews  Analyzed by reflexive thematic analysis | Three themes identified (each with thee subthemes)  1.distress to deliver treatment against consent (e.g. NG-feeding):  -feeling as a “punisher” instead a helper  -uncertainty if one helps or hurts (best approach in complex case?)  -staff burden (confronted with self-harm, suicidality, high mortality rates)  2. feeling abused:  -(verbal) aggression, being pushed back  - lack of acknowledgement and support  -service problems with insufficient staffing and training  3. Coping:  -adjusting mindset and expectations (e.g. with regard to recovery)  -rewarding aspect of relational work with a difficult to reach group (possible with long inpatient stays)  -exchange, supervision, being able to express oneself in the team | Trigger:  -no commitment to treatment  -comorbidity (trauma, autism)  -structure of the service |  | X | X |  | X |
| 9.  Tragantzo­poulou & Giannouli 2023 | N = 7  86% female  psychotherapists (professional background?)  $\geq$ 5 yrs of experience  57.1 % CBT  28,6% family therapy  14.3% person-centered therapy | Vulnerabilities and challenges when working with AN | Greece | AN  Adolescents? | Interviews  Analyzed by interpretative phenomenological analysis (IPA) | Two themes:  Therapist-related factors  (vulnerability; therapist bias)  Communication (tests of trust, parents and therapy involvement)  Feelings of  -inadequacy and despair when interventions are ineffective  -responsibility and distress, fear (that patients might discontinue treatment)  - fear of not being able to help | Vulnerability:  -Treating AN is demanding  -questioning own abilities as a therapist; uncertainty in treatment selection  -pressure to provide help  -pressure to be careful with words  - elevated sense of responsibility  -fear not being able to help  Therapeutic bias:  -assumption that AN is difficult to treat, patients are resistant and non-responsive to treatment, demand control, are afraid of maturity  Tests of trust:  -patients were experienced as cautious and hesitant to open up, sometimes lying, but trying to rely on someone  -difficulty to achieve trust  Parents/therapy involvement:  -difficulties to get parents involved, parents denial of illness  -patients are hesitant to involve parents |  | X | X | X |  |

M = mean; N = number; yrs = years; CT = countertransference; AN = anorexia nervosa; BN = bulimia nervosa; AN-BP = binge-purging type of AN; BMI = body mass index; NG = nasogastric-tube feeding

## = included in the previous review of Thompsen-Brenner et al. 2012

** = included in the review of Forget et al. 2011
