# Supplementary material for: Countertransference in the treatment of patients with eating disorders
Source: J Eat Disord. 2025 Oct 27;13:240. doi: 10.1186/s40337-025-01439-z (PMC12560542; doi:10.1186/s40337-025-01439-z)
Supplement: Supplementary file 3 — Supplementary Material 3 [file 40337_2025_1439_MOESM3_ESM.docx]

**Supplement 3: Excluded studies**

| **Publication** | **Reason for exclusion** | **Included in Thompson-Brenner et al. 2012** | **Included in Forget et al. 2011** |
| --- | --- | --- | --- |
| Blum (1987) [1] | Not on CT | Yes | No |
| Blum & Bearinger (1990) [2] | Not on CT | Yes | No |
| Gurney & Halmi (2001) [3] | Not on CT | Yes | No |
| Boulé & McSherry (2002) [4] | Not on CT | Yes | No |
| Loeb et al. (2005) [5] | Not on CT (on therap.alliance) | Yes | No |
| Friedman et al. (2008) [6] | Not on CT | Yes | No |
| Abbate-Daga et al.(2013) [7] | Not on CT | No | No |
| Thompson-Brenner (2014) [8] | Not on CT | No | No |
| Daly (2016) [9] | Not on CT | No | No |
| Gustafsson et al. (2021) [10] | Not on CT | No | No |
| Corral-Liria et al. (2022) [11] | Not on CT | No | No |
| Takimoto (2022) [12] | Not on CT | No | No |
| Wenn et al. (2002) [13] | Not on CT | No | No |
| Kiser (2023) [14] | Not on CT | No | No |
| Lachal et al. (2023) [15] | Not on CT | No | No |
| Ali (2023) [16] | Not on CT (on therap. alliance) | No | No |
| Albano et al. (2024) [17] | Not on CT | No | No |
| Silbiger et al. (2024) [18] | Not on CT | No | No |
| Barr et al. (2024) [19] | Not on CT (on therap. alliance) | No | No |
| Todd & Pine (1968) [20] | Not on EDs | No | No |
| Hayes et al. (1991) [21] | Not on EDs | No | No |
| Krimendahl (1994) [22] | Not on EDs | No | No |
| Krikorian & Fowler (2008) [23] | Not on EDs | No | No |
| Hayes et al. (2015) [24] | Not on EDs | No | No |
| Knaus et al. (2016) [25] | Not on EDs | No | No |
| Hayes et al. (2018) [26] | Not on EDs | No | No |
| Nissen-Lie et al. (2022) [27] | Not on EDs | No | No |
| Gelso & Kline (2022) [28] | Not on EDs | No | No |
| Colli et al. (2022) [29] | Not on EDs | No | No |
| Tishby & Wiseman (2022) [30] | Not on EDs | No | No |
| Costin & Johnson (2002) [31] | On therapists with own ED experience | No | No |
| Warren et al. (2013) [32] | On therapists with own ED experience | No | No |
| Chang et al. (2023) [33] | On therapists with own ED experience | No | No |
| Curry & Andriopoulou (2023) [34] | On therapists with own ED experience | No | No |
| King & Russon (2023) [35] | On therapists with own ED experience | No | No |
| Poiani-Cordella et al. (2024) [36] | On therapists with own ED experience | No | No |
| Bijkerk et al. (2024) [37] | On therapists with own ED experience | No | No |
| Bray et al. (2024) [38] | On therapists with own ED experience | No | No |
| Morgan (1977) [39] | Case report /theoretical discussion | No | Yes |
| Sohn (1985) [40] | Case report /theoretical discussion | No | No |
| Zerbe (1986) [41] | Case report/theoretical discussion | No | No |
| Birksted-Breen (1989)m[42] | Case report/theoretical discussion | No | Yes |
| Hamburg & Herzog (1990) [43] | Case report/theoretical discussion | No | Yes |
| Zunino et al. (1991) [44] | Case report/theoretical discussion | No | No |
| Baumann, (1992) [45] | Case report/theoretical discussion | No | No |
| Sarles (1994) [46] | Case report/theoretical discussion | No | No |
| Saloff-Coste et al. (1994) [47] | Case report/theoretical discussion | No | No |
| Zerbe (1995) [48] | Case report/theoretical discussion | No | No |
| Hughes (1997) [49] | Case report/theoretical discussion | No | Yes |
| Williams (1997)[50] | Case report/theoretical discussion | No | Yes |
| Russell & Marsden (1998) [51] | Case report/theoretical discussion | No | Yes |
| DeLucia-Waack (1999) [52] | Case report/theoretical discussion | No | No |
| Kaplan & Garfinkel 1999 [53] | Case report/theoretical discussion | No | Yes |
| Bulik & Kendler (2000) [54] | Case report/theoretical discussion | No | Yes |
| Strober (2004) [55] | Case report/theoretical discussion | No | Yes |
| Lowell & Meander (2005) [56] | Case report/theoretical discussion | No | No |
| Golan et al. (2009) [57] | Case report/theoretical discussion | No | No |
| Bunnell, (2016) [58] | Case report/theoretical discussion | No | No |
| Zerbe & Bradley (2018) [59] | Case report/theoretical discussion | No | No |
| Fixsen et al. (2023) [60] | Case report/theoretical discussion | No | No |
| Zerbe (1986) [61] | Other: Book chapter | No | No |
| Derenne (2006) [62] | Other: Report on experience when starting to work with EDs | No | No |
| Blackburn et al. (2021) [63] | Other: Focus on experiences with longstanding AN | No | No |
| Eckermann (2022) [64] | Other: Dissertation | No | No |
| Kanakam (2022) [65] | Other: Focus on ethnic minority females with EDs | No | No |
| Retkiewicz (2022) [66] | Other: Dissertation | No | No |
| Morgan & Firebaugh (2024) [67] | Other: Focus on treating EDs during COVID-19 pandemia | No | No |
| Ferrucci (2024) [68] | Other: Focus on gender minority patients | No | No |

**References**:

1. Blum R. Physicians’ assessment of deficiencies and desire for training in adolescent care. J Med Educ. 1987;62:401–7.

2. Blum RW, Bearinger LH. Knowledge and attitudes of health professionals toward adolescent health care. J Adolesc Health Care. 1990;11:289–94.

3. Gurney VW, Halmi KA. An eating disorder curriculum for primary care providers. Int J Eat Disord. 2001;30:209–12.

4. Boulé CJ, McSherry JA. Patients with eating disorders. How well are family physicians managing them? Can Fam Physician. 2002;48:1807–13.

5. Loeb K, Wilson T, Labouvie E, Pratt E, Hayaki J, Walsh B, et al. Therapeutic alliance and treatment adherence in two interventions for bulimia nervosa: a study of process and outcome. J Consult Clin Psychol. 2005;73 (6):1097–107.

6. Friedman KE, Ashmore JA, Applegate KL. Recent experiences of weight-based stigmatization in a weight loss surgery population: psychological and behavioral correlates. Obesity. 2008;16 Suppl 2:S69-74.

7. Abbate-Daga G, Amianto F, Delsedime N, De-Bacco C, Fassino S. Resistance to treatment and change in anorexia nervosa: a clinical overview. BMC Psychiatry. 2013;13:294.

8. Thompson-Brenner H. Discussion of “Eating disorders and attachment: a contemporary psychodynamic perspective:” does the attachment model of eating disorders indicate the need for psychodynamic treatment? Psychodyn Psychiatry. 2014;42:277–84.

9. Daly SB. The intersubjective experience of the physical body in the clinical setting of eating disorders. Clinical Social Work Journal. 2016;44:47–56.

10. Gustafsson SA, Stenström K, Olofsson H, Pettersson A, Wilbe Ramsay K. Experiences of eating disorders from the perspectives of patients, family members and health care professionals: a meta-review of qualitative evidence syntheses. J Eat Disord. 2021;9:156.

11. Corral-Liria I, Alonso-Maza M, González-Luis J, Fernández-Pascual S, Becerro-de-Bengoa-Vallejo R, Losa-Iglesias M. Holistic nursing care for people diagnosed with an eating disorder: A qualitative study based on patients and nursing professionals’ experience. Perspectives in Psychiatric Care. 2022;58:840–9.

12. Takimoto Y. International comparison of physicians’ attitudes toward refusal of treatment by patients with anorexia nervosa: A case-based vignette study. J of EatDisord. 2022;10. Available from: http://www.redi-bw.de/db/ebsco.php/search.ebscohost.com/login.aspx%3fdirect%3dtrue%26db%3dpsyh%26AN%3d2022-76850-001%26site%3dehost-live

13. Webb H, Dalton B, Irish M, Mercado D, McCombie C, Peachey G, et al. Clinicians’ perspectives on supporting individuals with severe anorexia nervosa in specialist eating disorder intensive treatment settings. J Eat Disord. 2022;10. Available from: http://www.redi-bw.de/db/ebsco.php/search.ebscohost.com/login.aspx%3fdirect%3dtrue%26db%3dpsyh%26AN%3d2022-21318-001%26site%3dehost-live

14. Kiser ET. Understanding adherence to cognitive behavioral therapy (CBT) in clinicians who treat eating disorders: A self-determination theory approach. ProQuest Information & Learning; 2023. Available from: http://www.redi-bw.de/db/ebsco.php/search.ebscohost.com/login.aspx%3fdirect%3dtrue%26db%3dpsyh%26AN%3d2023-10933-253%26site%3dehost-live

15. Lachal J, Carretier E, Prevost C, Nadeau P-O, Taddeo D, Fortin M-C, et al. The experience of healthcare professionals treating adolescents with eating disorders in psychiatric and pediatric inpatient units for adolescents: A qualitative study. L’Encéphale: Revue de psychiatrie clinique biologique et thérapeutique. 2023;49:331–41.

16. Ali SA. A qualitative exploration of licensed professional counselors’ therapeutic relationships working with clients with body image concerns. ProQuest Information & Learning; 2023. Available from: http://www.redi-bw.de/db/ebsco.php/search.ebscohost.com/login.aspx%3fdirect%3dtrue%26db%3dpsyh%26AN%3d2023-55664-205%26site%3dehost-live

17. Albano G, Teti A, Scrò A, Bonfanti RC, Fortunato L, Lo Coco G. A systematic review on the role of therapist characteristics in the treatment of eating disorders. Research in Psychotherapy: Psychopathology, Process and Outcome [Internet]. 2024;27. Available from: http://www.redi-bw.de/db/ebsco.php/search.ebscohost.com/login.aspx%3fdirect%3dtrue%26db%3dpsyh%26AN%3d2025-10658-001%26site%3dehost-live

18. Silbiger K. Mental health providers’ perceptions of restrictive eating disorders: Relationship with client body weight. Int J Eat Disord. 2024;57:916–23.

19. Barr BL, McIntosh VVW, Britt EF, Jordan J, Carter JD. Clinical factors and early life experiences associated with therapeutic alliance development in treatment for depression or binge eating. Psychother Res. 2024;34:4–16.

20. Todd WE, Pine I. Peer supervision of individual psychotherapy. Am J Psychiatry. 1968;125:780–784.

21. Hayes JA, Gelso CJ, Van Wagoner SL, Diemer RA. Managing countertransference: what the experts think. Psychol Rep. 1991;69:139–48.

22. Krimendahl EK. Review of Countertransference in psychotherapy with children and adolescents. Psychoanalytic Psychology. 1994;11:419–22.

23. Krikorian SE, Fowler JC. A view from Riggs: treatment resistance and patient authority--VII. A team approach to treatment resistance. J Am Acad Psychoanal Dyn Psychiatry. 2008;36:353–73.

24. Hayes JA, Nelson DLB, Fauth J. Countertransference in successful and unsuccessful cases of psychotherapy. Psychotherapy (Chic). 2015;52:127–33.

25. Knaus S, Grassl R, Seidman C, Seitz T, Karwautz A, Löffler-Stastka H. Psychiatrists’ emotional reactions: Useful for precise diagnosis in adolescence? Bull Menninger Clin. 2016;80:316–25.

26. Hayes JA, Gelso CJ, Goldberg S, Kivlighan DM. Countertransference management and effective psychotherapy: Meta-analytic findings. Psychotherapy (Chic). 2018;55:496–507.

27. Nissen-Lie HA, Dahl H-SJ, Høglend PA. Patient factors predict therapists’ emotional countertransference differently depending on whether therapists use transference work in psychodynamic therapy. Psychother Res. 2022;32:3–15.

28. Gelso CJ, Kline KV. Some directions for research and theory on countertransference. Psychother Res. 2022;32:59–64.

29. Colli A, Gagliardini G, Gullo S. Countertransference responses mediate the relationship between patients’ overall defense functioning and therapists’ interventions. Psychother Res. 2022;32:45–58.

30. Tishby O, Wiseman H. Countertransference types and their relation to rupture and repair in the alliance. Psychother Res. 2022;32:29–44.

31. Costin C, Johnson CL. Been there, done that: clinicians’ use of personal recovery in the treatment of eating disorders. Eat Disord. 2002;10:293–303.

32. Warren CS, Schafer KJ, Crowley MEJ, Olivardia R. Treatment providers with a personal history of eating pathology: a qualitative examination of common experiences. Eat Disord. 2013;21:295–309.

33. Chang Y-S, Liao F-T, Huang L-C, Chen S-L. The Treatment Experience of Anorexia Nervosa in Adolescents from Healthcare Professionals’ Perspective: A Qualitative Study. Int J Environ Res Public Health. 2023;20:794.

34. Curry EE, Andriopoulou P. “Dual-experiences” of treatment for anorexia nervosa: An interpretative phenomenological analysis of experiences of treatment by service providers who are recovered service users. Mental Health Review Journal. 2023;28:396–413.

35. King AA, Russon JM. “Bringing and removing self from the table”: Therapists’ use and management of eating disorder lived experience in the treatment of clients with eating disorders. Journal of Marital and Family Therapy. 2023;49:654–74.

36. Poiani-Cordella C, Toh WL, Phillipou A. Eating behaviours and personality characteristics of clinicians and researchers working in eating disorders. Eating Disorders: The Journal of Treatment & Prevention. 2024;32:195–211.

37. Bijkerk CA, Nooteboom LA, de Beer CB, de Vos JA, Vermeiren RRJM. Treating eating disorders by professionals with similar experiences: A systematic review. Eur Eat Disord Rev. 2024;32:963–79.

38. Bray M, Heruc G, Evans L, Wright ORL. The imperative of collaboration: Lived experience perspectives on team approaches in outpatient eating disorder treatment. Int J Eat Disord. 2024;57:116–23.

39. Morgan HG. Fasting girls and our attitudes to them. Br Med J. 1977;2:1652–5.

40. Sohn L. Anorexic and bulimic states of mind in the psycho-analytic treatment of anorexic/bulimic patients and psychotic patients. Psychoanalytic Psychotherapy. 1985;1:49–56.

41. Zerbe DH. Countertransference, resistance and frame management in the psychotherapy of a 15 year old anorexic and her mother. Clin Soc Work J. 1986;14:213–23.

42. Birksted-Breen D. Working with anorexic patient. Int J Psychoanal. 1989;70 ( Pt 1):29–40.

43. Hamburg P, Herzog D. Supervising the therapy of patients with eating disorders. Am J Psychother. 1990;44:369–80.

44. Zunino N, Agoos E, Davis WN. The impact of therapist gender on the treatment of bulimic women. Int J Eat Disord. 1991;10:253–63.

45. Baumann J. Reflections on group psychotherapy with eating-disordered patients. Group. 1992;16:95–100.

46. Sarles RM. Transference-countertransference issues with adolescents: personal reflections. Am J Psychother. 1994;48:64–74.

47. Saloff-Coste CJ, Hamburg P, Herzog DB. New dimensions for the dietitian in the treatment of patients with eating disorders. Eat Disord. 1994;2:47–56.

48. Zerbe KJ. Integrating feminist and psychodynamic principles in the treatment of an eating disorder patient: implications for using countertransference responses. Bull Menninger Clin. 1995;59:160–76.

49. Hughes P. The use of the countertransference in the therapy of patients with anorexia nervosa. Eur Eat Disord Rev. 1997;5:258–69.

50. Williams G. Reflections on some dynamics of eating disorders: “no entry” defences and foreign bodies. Int J Psychoanal. 1997;78 ( Pt 5):927–41.

51. Russell G, Marsden P. What does the therapist feel? Countertransference with bulimic women with borderline personality disorder. Br J Psychotherapy. 1998;15:31–42.

52. DeLucia-Waack JL. Supervision for counselors working with eating disorders groups: Countertransference issues related to body image, food, and weight. Journal of Counseling & Development. 1999;77:379–88.

53. Kaplan AS, Garfinkel PE. Difficulties in treating patients with eating disorders: a review of patient and clinician variables. Can J Psychiatry. 1999;44:665–70.

54. Bulik CM, Kendler KS. “I am what I (donït) eat”: establishing an identity independent of an eating disorder. Am J Psychiatry. 2000;157 (11):1755–60.

55. Strober M. Managing the chronic, treatment-resistant patient with anorexia nervosa. Int J Eat Disord. 2004;36:245–55.

56. Lowell MA, Meader LL. My Body, Your Body: Speaking the Unspoken between the Thin Therapist and the Eating-Disordered Patient. Clinical Social Work Journal. 2005;33:241–57.

57. Golan M, Yaroslavski A, Stein D. Managing eating disorders. Countertransference and other dynamic processes in the therapeutic milieu. International Journal of Child Health and Adolescent Health. 2009;2;213-227.

58. Bunnell D. Gender Socialization, Countertransference and the Treatment of Men with Eating Disorders. Clinical Social Work Journal. 2016;44.

59. Zerbe KJ, Bradley KM. Bring Me Your Hungers: Omnipotence, Mourning, and the Inexorable Limits of Time and Self in the Psychodynamic Treatment of Eating Disorders. Psychoanal Rev. 2018;105:363–95.

60. Fixsen A, Ridge D, Ponsford O, Holder M, Saran G. Battles over ‘unruly bodies’: Practitioners’ interpretations of eating disorders and the utility of psychiatric labelling. Sociology of Health & Illness. 2023;45:560–79.

61. Zerbe KJ. The emerging sexual self of the patient with an eating disorder: Implications for treatment. In: Schwartz MF, Cohn L, editors. Sexual abuse and eating disorders [Internet]. Philadelphia, PA: Brunner/Mazel; 1996 [cited 2021 Oct 21]. p. 134–54. Available from: http://www.redi-bw.de/db/ebsco.php/search.ebscohost.com/login.aspx%3fdirect%3dtrue%26db%3dpsyh%26AN%3d1996-97492-010%26site%3dehost-live

62. Derenne JL. The therapist’s voice: Junior High, revisited. Eat Disord. 2006;14:335–9.

63. Blackburn B, O’Connor J, Parsons H. Becoming needless: A psychoanalytically informed qualitative study exploring the interpersonal and intrapsychic experiences of longstanding anorexia nervosa. International Journal of Applied Psychoanalytic Studies. 2021;18:428–42.

64. Eckermann ED. A national study of medical professionals’ attitudes toward eating disorders. ProQuest Information & Learning; 2022. Available from: http://www.redi-bw.de/db/ebsco.php/search.ebscohost.com/login.aspx%3fdirect%3dtrue%26db%3dpsyh%26AN%3d2022-56509-020%26site%3dehost-live

65. Kanakam N. Therapists’ experiences of working with ethnic minority females with eating disorders: A qualitative study. Culture, Medicine, and Psychiatry: An International Journal of Cross-Cultural Health Research. 2022;46:414–34.

66. Retkiewicz E. Healthcare professionals’ capacity for compassion and interactions with people diagnosed with eating disorders. ProQuest Information & Learning; 2022. Available from: http://www.redi-bw.de/db/ebsco.php/search.ebscohost.com/login.aspx%3fdirect%3dtrue%26db%3dpsyh%26AN%3d2022-56509-272%26site%3dehost-live

67. Morgan SM, Mace Firebaugh C. Psychodynamic therapists treating patients with eating disorders during COVID-19: Perceptions of the therapeutic relationship, patient experiences and symptomatology, and therapeutic processes. Psychoanalytic Psychotherapy. 2024;38:153–66.

68. Ferrucci KA, Lapane KL, Jesdale BM, McPhillips E, Dubé CE. Eating disorder specialist views on gender competency and education for treating gender minority patients. The Journal of Behavioral Health Services & Research. 2024;51:232–49.
